# Validation of the Calypso Surface Beacon Transponder

**DOI:** 10.1120/jacmp.v17i4.6152

**Published:** 2016-07-08

**Authors:** Maxwell Belanger, Ziad Saleh, Tom Volpe, Rich Margiasso, Xiang Li, Maria Chan, Xiaofeng Zhu, Xiaoli Tang

**Affiliations:** ^1^ Applied Physics and Applied Mathematics, Columbia University New York NY; ^2^ Department of Human Oncology University of Wisconsin Madison Madison WI; ^3^ Department of Medical Physics Memorial Sloan Kettering Cancer Center West Harrison NY; ^4^ Department of Medical Physics Memorial Sloan Kettering Cancer Center Rockville Center NY; ^5^ Department of Medical Physics Memorial Sloan Kettering Cancer Center Basking Ridge NJ; ^6^ Department of Radiation Oncology University of Nebraska Medical Center Omaha NE USA

**Keywords:** radiotherapy, Calypso, external beam treatment, Surface Beacon

## Abstract

Calypso L‐shaped Surface Beacon transponder has recently become available for clinical applications. We herein conduct studies to validate the Surface Beacon transponder in terms of stability, reproducibility, orientation sensitivity, cycle rate dependence, and respiratory waveform tracking accuracy. The Surface Beacon was placed on a Quasar respiratory phantom and positioned at the isocenter with its two arms aligned with the lasers. Breathing waveforms were simulated, and the motion of the transponder was tracked. Stability and drift analysis: sinusoidal waveforms (200 cycles) were produced, and the amplitudes of phases 0% (inhale) and 50% (exhale) were recorded at each breathing cycle. The mean and standard deviation (SD) of the amplitudes were calculated. Linear least‐squares fitting was performed to access the possible amplitude drift over the breathing cycles. Reproducibility: similar setting to stability and drift analysis, and the phantom generated 100 cycles of the sinusoidal waveform per run. The Calypso system's was re‐setup for each run. Recorded amplitude and SD of 0% and 50% phase were compared between runs to assess contribution of Calypso electromagnetic array setup variation. Beacon orientation sensitivity: the Calypso tracks sinusoidal phantom motion with a defined angular offset of the beacon to assess its effect on SD and peak‐to‐peak amplitude. Rate dependence: sinusoidal motion was generated at cycle rates of 1 Hz, .33 Hz, and .2 Hz. Peak‐to‐peak displacement and SDs were assessed. Respiratory waveform tracking accuracy: the phantom reproduced recorded breathing cycles (by volunteers and patients) were tracked by the Calypso system. Deviation in tracking position from produced waveform was used to calculate SD throughout entire breathing cycle. Stability and drift analysis: Mean amplitude ± SD of phase 0% or 50% were 20.01±0.04 mm and ‐19.65±0.08 mm, respectively. No clinically significant drift was detected with drift measured as $5.1\times 10^{‐5}\;\text{mm}/s$ at phase 0% and $‐6.0\times 10^{‐5}\;\text{mm}/s$ at phase 50%. Reproducibility: The SD of the setup was 0.06 mm and 0.02 mm for phases 0% and 50%, respectively. The combined SDs, including both setup and intrarun error of all runs at phases 0% and 50%, were 0.07 mm and 0.11 mm, respectively. Beacon orientation: SD ranged from 0.032 mm to 0.039 mm at phase 0% and from 0.084 mm to 0.096 mm at phase 50%. The SD was found not to vary linearly with Beacon angle in the range of 0° and 15°. A positive systematic error was observed with amplitude 0.07 mm/degree at phase 0% and 0.05 mm/degree at phase 50%. Rate dependence: SD and displacement amplitudes did not vary significantly between 0.2 Hz and 0.33 Hz. At 1 Hz, both 0% and 50% amplitude measurements shifted up appreciably, by 0.72 mm and 0.78 mm, respectively. As compared with the 0.33 Hz data, SD at phase 0% was 1.6 times higher and 5.4 times higher at phase 50%. Respiratory waveform tracking accuracy: SD of 0.233 mm with approximately normal distribution in over 134 min of tracking (201468 data points). The Surface Beacon transponder appears to be stable, accurate, and reproducible. Submillimeter resolution is achieved throughout breathing and sinusoidal waveforms.

PACS number(s): 87.50.ct, 87.50.st, 87.50.ux, 87.50.wp, 87.50.yt

## I. INTRODUCTION

**Figure 1:**
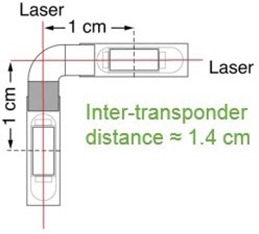
Schematic diagram showing the design of Calypso Surface Beacon.^(6)^

The Calypso Prostate Beacon transponder system (Varian Medical Systems Inc., Palo Alto, CA) has been widely used in the clinic. Varian Medical Systems has recently released an L‐shaped Surface Beacon transponder (Fig. 1) for real time tracking with the Calypso system. The Surface Beacon is comprised of two prostate transponders, with one high frequency and one intermediate frequency. The ability to track a nonsite‐specific, externally placed Beacon allows for noninvasive tracking of deep inhalation breath‐hold (DIBH) and free breathing, as well as general patient motion. Calypso system positioning has been previously assessed for static accuracy and precision,^1^, ^2^, ^3^ as well as the influence of motion on tracking accuracy.^(4)^ Although previous work has demonstrated the Calypso 4D tracking system provides greater accuracy than alternative tracking methods,^(5)^ these previous studies focus on tracking of three implanted Beacons. At the time of writing, no studies are available evaluating the Calypso Surface Beacon. We herein conduct studies to validate the Calypso Surface Beacon^(6)^ transponders. The Quasar phantom (Modus Medical Devices Inc., London, Canada) is able to reproduce the recorded volunteer and patient breathing patterns. One novel experiment we conducted was placing the Surface Beacon on the Quasar phantom so that we can study the real respiratory waves retrospectively.

## II. MATERIALS AND METHODS

### A. Calypso system

**Figure 2:**
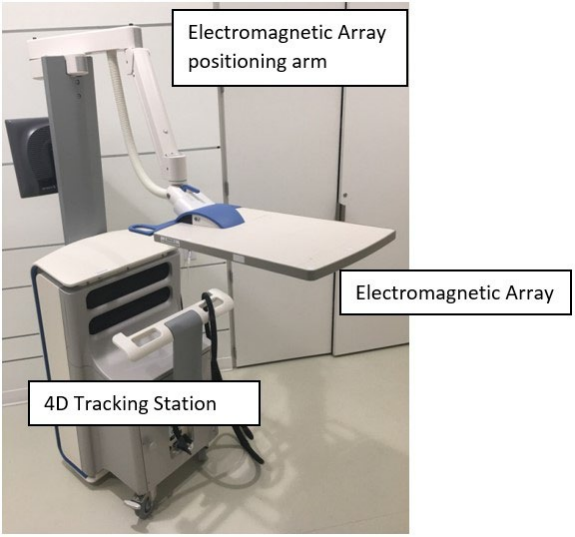
Calypso tracking station.

Detailed reports on the operational mechanics of the Calypso system (Fig. 2) have been reported^(7)^ and are briefly reproduced here. The Calypso system utilizes the electromagnetic signals to achieve precise and accurate tracking in real time. There are two major components — the beacon transponders and the electromagnetic array. The transponders are small (1.85×8.00 mm) devices powered by resonant frequencies of 300, 400, or 500 kHz from a source array. When energized, the transponder emits a response signal which may be localized relative to an array of sensor coils located in an electromagnetic array placed above isocenter. The electromagnetic array's position relative isocenter is established by an array of three infrared cameras, thus allowing for measurement of Beacon position relative to accelerator isocenter. The Surface Beacon varies from the implanted Beacons only in the number of transponders — two transponders in the Surface Beacon versus three in implantation. Sequential excitation and localization may be achieved at up to 25 Hz in the most recent Calypso models. Spatial resolution is expected to be on the order of 10‐^1^ mm.^(1)^

### B. Quasar respiratory phantom

**Figure 3:**
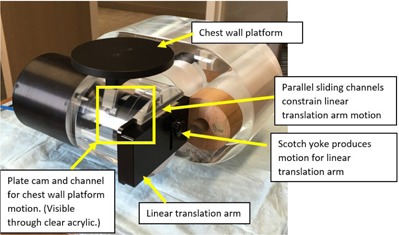
Quasar respiratory phantom in upright position.

All waveforms generated in this study were created using the Quasar respiratory phantom (Fig. 3). The Quasar respiratory motion phantom is a programmable state‐of‐the‐art breathing simulator. It is capable of not only producing sinusoidal motion at variable amplitudes (up to ±20 mm), but also reproducing recorded patient breathing waveforms (of amplitudes up to ±15 mm). The resolution (how fine the motion is) of the phantom motion is controlled by an internal optical encoder. It produces position measurements with a resolution of ±0.01 mm at a sampling rate of 100 Hz. The position and temporal resolution of the phantom encoder position measurement are approximately $\pm 1\times 10^{‐2}\;\text{mm}$ and $\pm 5\times 10^{‐3}\;s$, respectively. This resolution is greater than that of the Calypso system — it allows the phantom measurement to be used as the ground truth, and the motion deviation of Beacon can be calculated.

### C. Phantom setup

**Figure 4:**
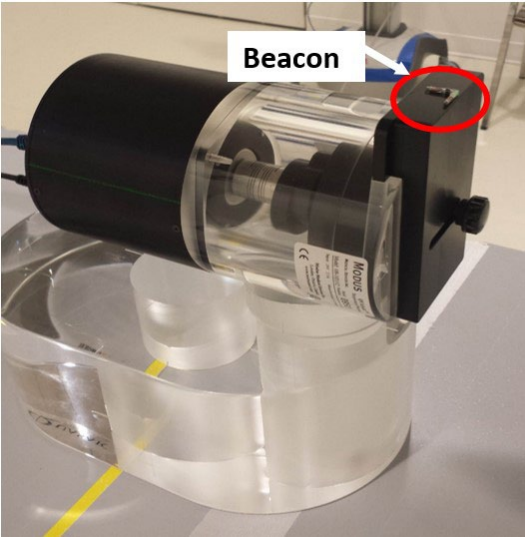
Phantom setup and Beacon (in red circle) placement. Beacon location relative the linear displacement arm is restrained by 3M Micropore tape. Tape is not depicted for clarity of Beacon position.

The phantom was set on its side with the Beacon placed on its linear translation arm such that the Beacon 0° angle aligned as measured by the Calypso coincided with 0° couch angle (Fig. 4). The Beacon is restrained relative to the phantom linear translation arm by 3M (Mapelwood, MN) Micropore tape. Placing the phantom on its side and use the linear translation arm rather than the chest wall platform allowed greater maximum displacement. The linear translation arm motion is produced by a Scotch yoke (Fig. 3) and constrained by two parallel sliding channels. In contrast, the chest wall platform motion is produced by a plate cam and constrained by a significantly looser channel. Thus the chosen setup is also able to significantly reduce off‐axis motion as compared to a traditional, upright, orientation of this phantom (Fig. 3). The Beacon, resting on the phantom, was placed at the isocenter with arms aligned to the transverse and sagittal axis/lasers. For the baseline setup, the phantom produced 0.2 Hz sinusoidal waves in the vertical axis with a peak‐to‐peak amplitude of 40.0±0.1 mm. Modifications to the baseline setup are outlined in their respective sections.

**Figure 5:**
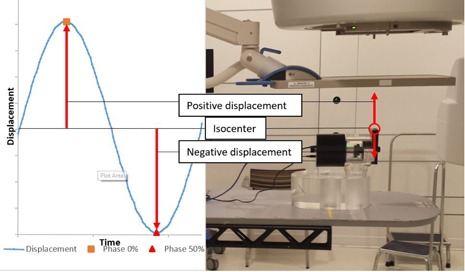
Beacon setup at isocenter, 0.00 mm displacement. Displacement in anterior direction relative isocenter taken as positive. Displacement in posterior direction relative isocenter taken as negative.

This setup produced motion on the anterior–posterior axis with displacement measured relative to accelerator isocenter along this axis (anterior and posterior amplitudes) (Fig. 5). Ground truth position measurement is taken by the phantom's on‐board optical encoder which reports position, as well as a time stamp. Beacon positioning is reported relative to isocenter along the anterior–posterior axis in the accelerators frame of reference.

### D. Recorded breathing files for respiratory phantom

Recorded breathing files were sourced from recordings of volunteers (6 of 37) and patients (13 of 37) and preprogrammed breathing sets from the Calypso phantom (18 of 37). Volunteer recorded breathing was measured using the Real‐Time Position Management (RPM) system (Varian Medical Systems Inc.). Breathing types included DIBH (16 of 37), normal breathing (11 of 37), irregular breathing (8 of 37), and deep exhalation breath‐hold (2 of 37). Waveforms with peak‐to‐peak displacement greater than 30 mm were scaled to 30 mm due to phantom mechanical restrictions.

### E. Data recording, fitting, and analysis

#### E.1 Stability and drift analysis

Sinusoidal motion using the baseline setup was recorded for 200 cycles (16.67 min). Mean displacement and standard deviation (SD) were calculated at phases 0% (inhale or maximal positive displacement) and 50% (exhale or maximal negative displacement). Linear least‐squares (LSQ) fitting was performed on positions at phase 0% and 50% to inspect possible systematic drift with time. The LSQ method finds the line of best fit by minimizing the sum of the square deviation between a data point and the fit line. Reduced chi‐squared, Eq. (1), is a measure of goodness of fit. In Eq. (1), *x* is the value of an experimental measure, μ is the value of the measure as predicted by the model in question, σ is the standard deviation of the distribution, *v* is the number of experimental measures taken, and *d.o.f*. represents the number of degrees of freedom of the sample. An ideal reduced chi‐squared value is 1 with a near zero value indicating that the error estimates are larger than actual and a high value indicating that the model is likely invalid. Reduced chi‐squared values are included given as an indication of the validity of the linear model.

(1) $$\chi_{reduced}^{2}=\frac{\sum_{i=1}^{v}\frac{{\left(x_{i}-\mu_{i}\right)}^{2}}{\sigma_{i}^{1}}}{d.o.f.}$$

#### E.2 Reproducibility

Using the baseline setup, five runs of 100 cycles were recorded with the Calypso. The Calypso system's electromagnetic array was repositioned to within manufacturer stated tolerance of ±5 mm and ±2° between each run. Phantom position remained unchanged between runs. Mean displacement and SD were calculated at phase 0% and 50% for comparison between runs. Net SD was calculated for all runs and compared with SD found in stability test to assess the impact of typical variation in electromagnetic array position on system accuracy.

#### E.3 Beacon orientation

Four runs of 100 cycles were recorded with the Calypso at Beacon angles of 0°, 5°, 10°, and 15°. The position of the electromagnetic array was unchanged between each run. The phantom/transponder angle was introduced by rotating the couch isocentrically. Beacon angle was determined by couch angle and compared to Calypso measured angle. Mean displacement and SD were calculated at phases 0% and 50% for comparison between runs.

#### E.4 Rate dependence

The phantom produced sinusoidal motion for 100 cycles at rates of 0.2 Hz, 0.33 Hz, and 1 Hz^†^. Neither the Calypso system's position nor the phantom's positions were changed between runs. Mean displacement and SD were calculated at phases 0% and 50% and were compared between runs.

#### E.5 Real respiratory waveform tracking accuracy

The Calypso system, Beacon, and Quasar phantom were set up as in the baseline setup, but the phantom was made to produce recorded breathing waveforms of volunteers and patients. Positioning measurements were exported from the Calypso and the Quasar phantom following each dataset.

Using the time stamps, the Calypso system and the Quasar phantom were synchronized. Fitting of the time information was required as the Calypso and Quasar phantom internal reference time could not be synchronized. This was accomplished, correcting all times with an offset, a constant time added or subtracted from each time stamp, such that the initial sample is taken at time t=0.00 s and the spacing between time stamps is preserved. An amplitude offset and multiplier were then applied to the Calypso measurement to account for registration mismatch and setup uncertainties. Maximum agreement between Calypso and phantom position measurements was achieved by minimizing the sum of the point‐wise square deviation between the Calypso and phantom position as measured by integrated optical encoder. The generalized reduced gradient (GRG) nonlinear solving method was used to optimize the position and timing fit parameters. GRG is one of the best deterministic local optimization methods. It is a gradient‐based method always looks for optimum closest to the starting point whether it is a local or global one. Deviation from fit was recorded for all runs and net SD was calculated. Equation (2) describes this fitting process where *Δt* is the time offset, *a* is the amplitude offset, and *b* is the amplitude multiplier. The form of this correction was chosen based on the setup variables of the phantom which included centering the center of phantom motion with isocenter, offset, and amplitude scaling by anterior‐posterior axis alignment and phantom Scotch yoke slide pin positioning, multiplier. The average magnitude offset value, a, was ‐0.41 mm and the average magnitude multiplier was 0.99. Values of Δt vary significantly between runs because of differences in the start of recording time.

$$\begin{array}{l} t^{Phantom,corrected}=t^{Phantom,raw}+\Delta t\\ d^{Phantom,corrected}=a+b\cdot d^{Phantom,raw} \end{array}$$

where *Δt, a*, and *b* are determined by minimizing the Sum of Square Deviation:

(2) $$Sum\;of\;Square\;Deviation=\sum_{i=1}^{v}{\left(d_{i}^{Calypso}-d_{i}^{Phantom,corrected}\right)}^{2}$$

## III. RESULTS

### A.1 Stability and drift analysis

**Figure 6:**
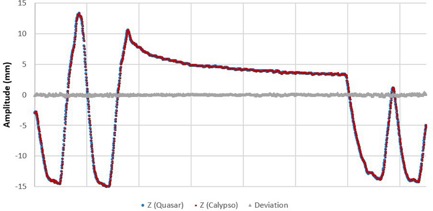
Sample DIBH trace as acquired by RPM system and captured by Calypso. Overlaid also is the residue of the LSQ fit of the two curves.

Mean displacement was recorded as 20.01±0.04 mm at phase 0% and ‐19.65±0.08 mm at phase 50%. The SDs at phases 0% and 50% are 0.04 mm and 0.08 mm, respectively. Observed average displacement deviated from produced waveform by 0.01 mm at phase 0% and 0.35 mm at phase 50% which is below the setup precision of the phantom, estimated to be ±0.1 mm. LSQ fits slope at phase 0% of $5.1\times 10^{‐5}\;\text{mm}/s$ with reduced chi‐squared of 0.72. LSQ fits slope at phase 50% of $‐6\times 10^{‐5}\;\text{mm}/s$ with reduced chi‐squared of 0.96 (see Fig. 6).

### A.2 Reproducibility

Average amplitude at phase 0% and 50% had SD of 0.06 mm and 0.02 mm respectively. Standard deviation of the composite dataset comprised of all reproducibility data at phase 0% and 50% was 0.065 mm and 0.116 mm, respectively. The expected values for combined error, as calculated by quadrature sum of electromagnetic array setup error and intrarun error, were found to be 0.070 mm at phase 0% and 0.113 mm at phase 50%.

### A.3 Beacon orientation

Beacon orientation is summarized in Table 1.

**Table 1:** Summary of Beacon displacement for different orientations at 0% and 50% phases

*Beacon Angle*	*Beacon Angle as Recorded by Calypso*	*Average Phase 0% Displacement (mm)*	*SD of Phase 0% Displacement (mm)*	*Average Phase 50% Displacement (mm)*	*SD of Phase 50% Displacement (mm)*
0	0	20.01	0.04	‐19.65	0.08
5.0	4.4	20.03	0.03	‐19.70	0.10
10.0	9.9	20.10	0.03	‐19.61	0.08
15.0	14.8	20.10	0.04	‐19.60	0.09

### A.4 Rate dependence

Beacon displacement as a function of breathing rates is summarized in Table 2.

**Table 2:** Summary of Beacon displacement as a function of breathing rates

*Cycle Rate (Hz)*	*Average Phase 0% Displacement (mm)*	*SD of Phase 0% Displacement (mm)*	*Average Phase 50% Displacement (mm)*	*SD of Phase 50% Displacement (mm)*
0.2	20.01	0.04	‐19.65	0.08
0.33	19.96	0.05	‐19.64	0.11
1	20.73	.08	‐18.87	0.59

### A.5 Respiratory waveform tracking accuracy

**Figure 7:**
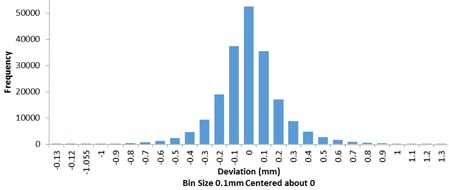
The distribution of the deviation which obeys an approximately normal distribution.

**Figure 8:**
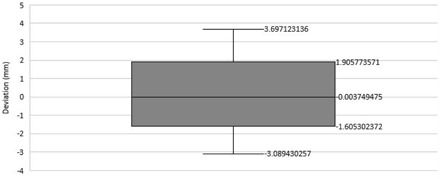
Box plot of deviation in measured Beacon position for respiratory waveform data.

Standard deviation of 0.233 mm with approximately normal distribution in over 134 min of tracking (201,468 data points). Median offset of ‐0.004 mm and skew of 0.02 are observed in deviation distribution (Fig. 7). Figure 8 gives the minimum, 25th percentile, median, 75th percentile, and maximum deviation observed in all breathing runs.

## IV. DISCUSSION

The Calypso system appears to be capable of consistently delivering submillimeter precision in a variety of tests. Although some amplitude drifts were detected in the stability test, the drift velocity was on the order of nanometers per second, which is unlikely to become clinically significant for even the longest treatment durations. The detected drift was in the superior direction for both 0% and 50% phase. Such a drift may have arisen from gravity‐driven downward motion of the electromagnetic array with smaller amplitude than was optically detectable by the Calypso system cameras. This possibility highlights the need to maintain the electromagnetic array arm which does not lock into position (Fig. 2).

Reproducibility measurements demonstrated that setup error is a contributing factor to system precision. The submillimeter values of net SD indicate that the system maintains a clinically useful measurement resolution even with the inclusion of accepted setup error. Interestingly, setup error was demonstrated to have a greater impact on the phase 0% cycle location than the phase 50% position. This trend may be explainable as the percent difference for the 0% phase, which places the Beacon maximally proximal to the electromagnetic array. Closer proximity at this phase would mean that any deviation in electromagnetic array to Beacon distance would have a higher percent error than the same deviation at phase 50%. This relationship is the opposite of the trend observed in intra‐run variance, to be discussed in a preceding paragraph, supporting the assertion that setup error is a distinct source of error.

The conclusion that setup error and intrarun error are mutually exclusive is further supported by the result that the sources of error added in quadrature yield approximately the SD found by a pointwise calculation on the composite dataset. The explanation of this result is as follows. The Calypso system's measurement relative to isocenter is actually a computation based on two distinct measurements. First, the position of the electromagnetic array is localized relative to isocenter by the camera array. Uncertainty in this localization is expected to be primarily responsible for setup error. The second measurement occurs when the electromagnetic array measures the location of the Beacon relative to itself. This process is expected to be the primary source of random intrarun error. It will be shown in the proceeding paragraphs that measurement deviation is not correlated with Beacon to electromagnetic array distance within the explored range. These two measurements are combined in software as a coordinate addition to yield Beacon position relative to isocenter. Propagation of error by partial derivatives results in net error equal to the quadrature addition of setup and intrarun error, which is the observed result. It is recommended that therapists take care to setup the electromagnetic array as consistently as possible, and that the site setup tolerances be lower than accepted by the manufacturer.

Beacon orientation was found not to yield a linear increase in SD. Linear LSQ fits of the data did yield positive trends of .07 mm/degree at phase 0% and 0.05 mm/degree at phase 50%. However, the high reduced chi‐squared values, 35.5 and 19.6 respectively, indicate that these fits are highly suspect. These trends may also have been the result of the system's longitudinal axis being imperfectly coincident with the couch's rotational axis. It is also worth noting that the Calypso measurement of Beacon angle was not in agreement with the calibrated couch angle measurement to which the Beacon was aligned. Although the angle measurements were all underestimated, the amplitude of the disagreement was not systematically consistent. This contrasts with some previous work on Calypso prostate transponder measurement which reported that the Calypso measured the angles as larger than actual.^(8)^ The Xu study concluded that small (>9 mm) intertransponder separations be avoided to reduce this uncertainty. The intertransponder distance for the surface Beacon is measured at 14 mm (Fig. 1). Inconsistency in amplitude of angle measurement disagreement strongly suggests that the error is within the Calypso's measure of Beacon angle because couch off axis couch rotation would be expected to yield a linear offset in the range of 0° to 15°.

An increase in SD was found only for the highest, 1 Hz, cycle rate. Even at this extremely high rate, the SD remained in the submillimeter range, 0.08 mm at phase 0% and 0.59 mm at phase 50%. Loss of resolution at high cycle rate disproportionately affected the phase 50% measurement as compared to the phase 0% measurement. At this rate, there was also a shift in measurement amplitude by +0.72 mm at phase 0% and ‐0.78 mm at phase 50%. The peak‐to‐peak amplitude only decreased by an average of 0.06 mm. The source of this shift is not understood, but it may have arisen due to increased electromagnetic noise from the phantom drive motor and/or some tracking error internal to the Calypso.

It should also be noted here that the actual sampling rate of the Calypso system dropped from an average of 24.1 Hz to 21.2 Hz for the 1 Hz measurement. One likely source of this reduction is the internal error elimination software, which prevents the system from recording erroneous measurements. This behavior is consistent with issues that have been seen in which electromagnetic noise arises from the phantom drive motor.

The mean SD observed for all sinusoidal data between 0.2 Hz and 0.33 Hz was 0.07 mm, while the maximum observed SD was 0.16 mm. Interestingly, the SD at 50% was found to be, on average, 2.67 times greater than at phase 0%. The one likely explanation for this is electromagnetic interference produced by the phantom's drive motor. At phase 50%, the Beacon is most proximal to the drive motor and metallic couplings. Another likely cause is the reduction in expected electromagnetic return due to the additional distance between electromagnetic array and Beacon. Comparing Calypso measurements to phantom encoder positioning, no correlation was found between displacement and deviation. This asymmetrical error source is small but uncertainty, as to its source, merits some additional exploration.

**Figure 9:**
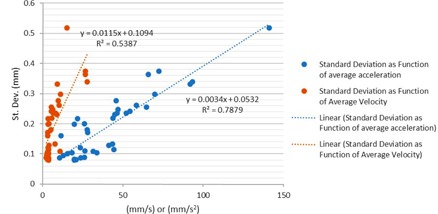
A strong, $R^{2}=0.79$, correlation was found between average absolute acceleration and SD. A weaker linear correlation, $R^{2}=0.54$, was also found between a run's average absolute velocity and SD.

System precision fell to an SD of 0.223 mm for the breathing waveform data. This finding is very robust as it is derived from the largest dataset with the most precise baseline measurement of which the authors are aware. The conclusion represents the ability of the system to track real breathing waveforms at every point in tracking. While no correlation was found between deviation and time, position, velocity, or acceleration, for a given run, a strong linear correlation, $R^{2}=0.79$, was found between the SD and average absolute acceleration (Fig. 9). A weaker linear correlation, $R^{2}=0.54$, was also found between the average absolute velocity and SD. It is important to note that the slope of this correlation is small for both average absolute acceleration ($0.003\;\text{mm}/(\text{mm}/s^{2})$) and average absolute velocity (0.012 mm/(mm/s)).

It is also worth noting that coincidence between Calypso measurement position and Beacon position was achieved by optimizing a linear fit. Although this allowed for precise measurement of SD, it inhibits the direct measurement of any offset in measurement. Previous work has shown disagreement between Calypso measurement and the implanted transponder, ranging from 1.1 mm^(9)^ to 1.5 mm.^(10)^ Uncorrected data yielded an average amplitude of correction of 0.32 mm. Given that the amplitude precision of the phantom setup is estimated to be ±0.1 mm; it is apparent that the agreement between this measure and the Calypso is superior to previously cited measures, which were based on X‐ray and CBCT measurement. However, the experimental design used here only made an absolute position measurement for the zero, isocenter, position. Other positions were calibrated by displacement from isocenter, making them susceptible to axis misalignment. Thus, the observed threefold increase in positioning agreement should not be viewed as a definitive result due to the inability of the experimental design to give absolute positional measurements in three dimensions. Our study produces no motion tracking results in three dimensions or with rotation during motion. Although we have no reason to believe the results would vary when extended to three dimensions, this is only supposition based on subjective observation. There is, however, no evidence to suggest a clinically significant change in observed precision under three‐dimensional motion.

## V. CONCLUSIONS

The Surface Beacon transponder appears to be stable, accurate, and reproducible. Submillimeter resolution is achieved throughout breathing and sinusoidal waveforms. No positional drift was observed within runs and the standard deviation within a run was shown to be independent of displacement, velocity, and acceleration. Although Beacon angle offset was not found to correlate with SD, angular measurement was observed to deviate nonlinearly from true Beacon angle. It is recommended that attention be placed on optimizing electromagnetic array position as setup error has been shown to be a significant error contributor. Patients are advised to maintain as slow and steady a respiratory rate as possible to minimize the slight decrease in measurement resolution with increased transponder acceleration.

## ACKNOWLEDGMENTS

The authors acknowledge the MSK Cancer Center Support Grant/Core Grant (P30 CA008748) in partial support of this article.

## COPYRIGHT

This work is licensed under a Creative Commons Attribution 3.0 Unported License.

## References

[1] Balter JM , Wright JN , Newell LJ et al. Accuracy of a wireless localization system for radiotherapy. Int J Radiat Oncol Biol Phys. 2005;61(3):933–37. doi:10.1016/j.ijrobp.2004.11.009 15708277 10.1016/j.ijrobp.2004.11.009

[2] Santanam L , Noel C , Willoughby TR et al. Quality assurance for clinical implementation of an electromagnetic tracking system. Med Phys. 2009;36(8):3477–86. doi:10.1118/1.3158812 19746781 10.1118/1.3158812

[3] Franz AM , Schmitt D , Seitel A et al. Standardized accuracy assessment of the calypso wireless transponder tracking system. Phys Med Biol. 2014;59(22):6797–810. doi:10.1088/0031‐9155/59/22/6797 25332308 10.1088/0031-9155/59/22/6797

[4] Murphy MJ , Eidens R , Vertatschitsch E , Wright JN . The effect of transponder motion on the accuracy of the Calypso Electromagnetic Localization System. Int J Radiat Oncol Biol Phys. 2008;72(1):295–99. doi:10.1016/j. ijrobp.2008.05.036 18722280 10.1016/j.ijrobp.2008.05.036

[5] Williams E , Najib M . Assessing the net health outcomes of a 4D electromagnetic tumor tracking system during radiotherapy for clinically localized prostate cancer: a health technology assessment of the Calypso® 4D localization system with Beacon® Transponders. Value Heal. 2007;10(3):A123. doi:10.1016/S1098‐3015(10)68913‐9

[6] Varian Medical Systems . Calypso system surface transponder usage guide. Palo Alto, CA: Varian Medical Systems; 2013.

[7] Mate T , Krag D , Wright J , Dimmer S . A new system to perform continuous target tracking for radiation and surgery using non‐ionizing alternating current electromagnetics. Int Congr Ser. 2004;1268:425–30. doi:10.1016/j. ics.2004.03.209

[8] Xu Q , Li J , Shan G et al. Comparison of prostate rotation and Calypso beam rotation for prostate margin evaluation [abstract]. Med Phys. 2009;36(6):2704. doi:10.1118/1.3182259

[9] Mao W , Wang J , Foster R , Song K , Solberg T . Direct investigation of geometric coincidence among Calypso system, Onboard KV imaging, and MV treatment beam imaging [abstract]. Med Phys. 2010;37(6):3149. doi:10.1118/1.3468245

[10] Burch D , Willoughby T , Meeks S et al. Real time prostate translation, rotation, deformation evaluated with Calypso Beacon transponders. Int J Radiat Oncol. 2005;63(Suppl 1):S195. doi:10.1016/j.ijrobp.2005.07.338

